# Ear Sequelæ of Acute Diseases

**Published:** 1904-08-20

**Authors:** 


### EAR SEQUELÆ OF ACUTE DISEASES.

McKernon[1] points out the necessity of frequent otoscopic examinations of children suffering from acute diseases, which in the exanthemata he holds should be daily. He says that the difficulty in children would be greatly lessened were smaller specula used, and the auricle drawn downwards and backwards, instead of upward, backwards and outwards. At times the membrane shows no signs of congestion, but if it were brushed by a cotton-tipped probe exfoliatory epithelium would be brushed away, and it would be seen to be red and bulging. At other times the upper half looks normal save for Shrapnell's membrane, whereas the lower half looks dull and lustreless and of a greyish mottled appearance—this shows that the mucous membrane of the tube and middle ear is swollen and pouring forth a serous exudate. To save further trouble the inflammatory products should be evacuated at once by an opening through the membrane.

[1] Medical Record, June 25, 1904.

